# Supplementary material for: Pollination implications of the diverse diet of tropical nectar-feeding bats roosting in an urban cave
Source: PeerJ. 2018 Mar 26;6:e4572. doi: 10.7717/peerj.4572 (PMC5875395; doi:10.7717/peerj.4572)
Supplement: Supplemental Information 1 — Illumina adaptors are underlined whereas primer sequence are shown in regular font. [file peerj-06-4572-s001.docx]

Primers used in this study. Illumina adaptors are underlined whereas primer sequence are shown in regular font.

| **Target Amplicon** | **Direction** | **Illumina adaptor + Primer sequence (5’-3’)** | **References for Primers** |
| --- | --- | --- | --- |
| *ITS2*  (350bp) | Forward | TCGTCGGCAGCGTCAGATGTGTATAAGAGACAGATGCGATACTTGGTGTGAAT | Chen *et al.* (2010) |
| *ITS2*  (350bp) | Reverse | GTCTCGTGGGCTCGGAGATGTGTATAAGAGACAGTCCTCCGCTTATTGATATGC | White *et al.* (1990) |
| *rbcL*  (600bp) | Forward | TCGTCGGCAGCGTCAGATGTGTATAAGAGACAGATGTCACCACAAACAGAGACTAAAGC | Kress and Erickson (2007) |
| *rbcL*  (600bp) | Reverse | GTCTCGTGGGCTCGGAGATGTGTATAAGAGACAGAGTCCACCGCGTAGACATTCAT | de Vere *et al.* (2012) |

**Reference:**

Chen, S., Yao, H., Han, J., Liu, C., Song, J., Shi, L., ... & Luo, K. (2010). Validation of the ITS2 region as a novel DNA barcode for identifying medicinal plant species. *PLoS one*, 5(1), e8613.

de Vere, N., Rich, T. C., Ford, C. R., Trinder, S. A., Long, C., Moore, C. W., ... & Tatarinova, T. (2012). DNA barcoding the native flowering plants and conifers of Wales. *PLoS One*, 7(6), e37945.

Kress, W. J., & Erickson, D. L. (2007). A two-locus global DNA barcode for land plants: the coding rbcL gene complements the non-coding trnH-psbA spacer region. *PLoS One*, *2*(6), e508.

White, T. J., Bruns, T., Lee, S. J. W. T., & Taylor, J. W. (1990). Amplification and direct sequencing of fungal ribosomal RNA genes for phylogenetics. *PCR protocols: a guide to methods and applications, 18*(1), 315-322.
