# Supplementary material for: Pollination implications of the diverse diet of tropical nectar-feeding bats roosting in an urban cave
Source: PeerJ. 2018 Mar 26;6:e4572. doi: 10.7717/peerj.4572 (PMC5875395; doi:10.7717/peerj.4572)
Supplement: Supplemental Information 3 — R script used for estimating the species richness and sampling completeness ratio. [file peerj-06-4572-s003.docx]

#R script for estimating species richness and sampling completeness ratio

data <- read.csv("final.csv",row.names=1)

library(SpadeR)

#using Chao2 estimation (we cannot use abundance)

#we use frequency (absence and presence following suggestion from Prosser & Hebert 2017)

ChaoSpecies(data,datatype="incidence_raw",conf=0.95)

#rarefaction and extrapolation analyses

library(iNEXT)

library (ggplot2)

library(gridExtra)

ChaoRichness(data, datatype = "incidence_raw", conf = 0.95)

out<-iNEXT(data,q=0,"incidence_raw",se=TRUE,endpoint=52,conf=0.95, nboot=100)

#plot sample-size-based curve

p1<-ggiNEXT(out, type=1,se=T)+ xlab("Number of\nSampling Weeks")+ylab("Number of Plant Species")+scale_colour_manual(values=c("black"))+scale_fill_manual(values=c("gray25"))+ theme(panel.background = element_rect(fill = 'white', colour="gray19"),panel.grid.major = element_line(colour = "gray89"))

p11<-p1+ theme(plot.margin = unit(c(1,0.2,-1.1,1), "cm"))

#plot sample completeness curve

p2<-ggiNEXT(out, type=2, se=T)+ xlab("Number of\nSampling Weeks")+ylab("Sampling\nCompleteness Ratio")+scale_colour_manual(values=c("black"))+scale_fill_manual(values=c("gray25"))+ theme(panel.background = element_rect(fill = 'white', colour="gray19"),panel.grid.major = element_line(colour = "gray89"))

p22<-p2+ theme(plot.margin = unit(c(1,0.2,-1.1,0), "cm"))

grid.arrange(p11,p22,ncol=2,nrow=1)
