# Supplementary material for: Pollination implications of the diverse diet of tropical nectar-feeding bats roosting in an urban cave
Source: PeerJ. 2018 Mar 26;6:e4572. doi: 10.7717/peerj.4572 (PMC5875395; doi:10.7717/peerj.4572)
Supplement: Supplemental Information 4 — References: 1= Start & Marshall (1976) reported 31 plant species in diet of E. spealea roosting at Batu Caves and Gua Sanding in Peninsular Malaysia; 2= Hodgkison et al. (2004) reported four plant species in diet of E. spealea at primary forest in Peninsular Malaysia; 3= Bumrungsri et al. (2013) reported eleven plant species in diet of E. spelaea at Khao Kao Cave in Thailand; 4= Thavry et al. (2017) reported thirteen plant species in diet of E. spealea at Bat Khteas Cave in Cambodia; 5= This study detected 55 plant species using DNA metabarcoding. [file peerj-06-4572-s004.docx]

Checklist of plants consumed by *Eonycteris spelaea*. References: 1= Start & Marshall (1976) reported 31 plant species in diet of *E. spealea* roosting at Batu Caves and Gua Sanding in Peninsular Malaysia; 2= Hodgkison *et al.* (2004) reported four plant species in diet of *E. spealea* at primary forest in Peninsular Malaysia; 3= Bumrungsri *et al.* (2013) reported eleven plant species in diet of *E. spelaea* at Khao Kao Cave in Thailand; 4=Thavry *et al.* (2017) reported thirteen plant species in diet of *E. spealea* at Bat Khteas Cave in Cambodia; 5=This study detected 55 plant species using DNA metabarcoding.

| **Family** | **Species** | **Status** | **Type of detection^a^** | **References** |
| --- | --- | --- | --- | --- |
| Amaranthaceae | *Beta vulgaris* | Exotic | DNA | 5 |
|  | *Cyathula prostrata* | Native | DNA | 5 |
| Anacardiaceae | *Mangifera indica* | Exotic | DNA | 5 |
|  | *Mangifera* (Unidentified) |  | P | 1 |
| Apiaceae | *Cuminum cyminum* | Exotic | DNA | 5 |
|  | *Foeniculum vulgare* | Exotic | DNA | 5 |
| Araliaceae | *Schefflera* (Unidentified) |  | DNA | 5 |
| Arecaceaea | *Cocos nucifera* | Native | P | 1, 3 |
|  | *Arenga* (Unidentified) |  | P | 1, 3 |
|  | (Unidentified) |  | DNA | 5 |
| Asteraceae | *Bidens pilosa* | Native | DNA | 5 |
| Anacardiaceae | *Chrysanthemum* (Unidentified) | Exotic | DNA | 5 |
|  | *Mikania micrantha* | Exotic | DNA | 5 |
| Bignoniaceae | *Oroxylum indicum* | Native | P, DNA | 1, 3, 4, 5 |
|  | *Pajanelia longifolia* | Native | Fl | 1 |
| Cannabaceae | *Trema cannabina* | Native | DNA | 5 |
| Caricaceae | *Carica papaya* | Exotic | DNA | 5 |
| Compositae | (Unidentified) |  | P | 1 |
| Euphorbiaceae | *Croton argyratus* | Native | DNA | 5 |
|  | *Macaranga* (Unidentified) |  | DNA | 5 |
|  | *Mallotus paniculatus* | Native | DNA | 5 |
| Fabaceae | *Bauhinia strychnoidea* | Native | DNA | 5 |
|  | *Leucaena leucocephala* | Exotic | DNA | 5 |
| Gentianaceae | *Limahlania crenulata* | Native | DNA | 5 |
| Lamiaceae | *Vitex* (Unidentified) |  | DNA | 5 |
| Lecythidaceae | *Barringtonia* (Unidentified) |  | P | 1 |
| Leguminosae | *Parkia javanica* |  | Fl, P | 1, 2 |
|  | *Parkia speciosa* |  | Fl, P | 1, 2 |
|  | *Parkia singularis* |  | Fl | 1 |
|  | *Parkia* (Unidentified) |  | P | 1, 3, 4 |
| Lythraceae | *Duabanga grandiflora* | Native | Fl, P, DNA | 1, 5 |
|  | *Lagerstroemia speciosa* | Native | DNA | 5 |
|  | *Punica granatum* | Exotic | DNA | 5 |
|  | *Sonneratia alba* | Native | Fl, P | 1 |
|  | *Sonneratia caseolaris* | Native | Fl, P, DNA | 1, 5 |
|  | *Sonneratia ovata* | Native | Fl, P | 1 |
|  | *Sonneratia* (Unidentified) |  | P | 3, 4 |
| Malvaceae | *Bombax anceps* | Native | Fl, P | 1, 4 |
|  | *Bombax* (Unidentified) |  | P | 3 |
|  | *Ceiba pentandra* | Exotic | Fl, P, DNA | 1, 3, 4, 5 |
|  | *Durio zibethinus* | Native | Fl, P | 1, 3, 4 |
|  | *Durio graveolens* | Native | Fl | 1 |
|  | *Durio* (Unidentified) |  | P, DNA | 1, 5 |
| Moraceae | *Artocarpus elasticus* | Native | DNA | 5 |
|  | *Artocarpus heterophyllus* | Exotic | DNA | 5 |
|  | *Artocarpus* (Unidentified) |  | P | 1 |
|  | *Ficus benjamina* | Exotic | DNA | 5 |
|  | *Ficus calcicola* | Native | DNA | 5 |
|  | *Ficus* (Unidentified) |  | DNA | 5 |
| Musaceae | *Musa acuminata*  (previously reported as *malaccensis* and *truncata*) | Native | Fl, DNA | 1, 5 |
|  | *Musa balbisiana* | Native | DNA | 5 |
|  | *Musa* (Unidentified) |  | Fl, P, DNA | 1, 3, 4, 5 |
| Myrtaceae | *Syzygium jambos* | Native | DNA | 5 |
|  | *Syzygium malaccense*  (previously reported as *Eugenia malaccensis*) | Native | Fl | 1 |
|  | *Syzygium samarangense* | Exotic | DNA | 5 |
|  | *Syzygium* (Unidentified) |  | P | 1, 3 |
|  | *Xanthostemon chrysanthus* | Exotic | DNA | 5 |
|  | *Eucalyptus* (Unidentified) |  | P, DNA | 4, 5 |
| Piperaceae | *Piper aduncum* | Exotic | DNA | 5 |
| Rhizophoraceae | *Rhizophora* (Unidentified) |  | P | 1 |
| Rosaceae | *Pyrus* (Unidentified) |  | DNA | 5 |
| Rubiaceae | *Oldenlandia corymbosa* | Exotic | DNA | 5 |
|  | *Urophyllum leucophlaeum* | Native | DNA | 5 |
| Rutaceae | *Citrus* (Unidentified) |  | DNA | 5 |
| Sapindaceae | *Dimocarpus longan* | Native | DNA | 5 |
|  | *Nephelium ramboutan-ake* | Native | DNA | 5 |
| Sapotaceae | *Manilkara zapota* | Exotic | DNA | 5 |
|  | *Mimusops elengi* | Native | DNA | 5 |
|  | *Palaquium hispidum* | Native | Fl | 2 |
|  | *Palaquium obovatum* | Native | Fl, Fr | 2 |
|  | (Unidentified) |  | P | 1 |
| Zingiberaceae | *Etlingera* (Unidentified) |  | DNA | 5 |
| Athyriaceae | *Diplazium esculentum* | Native | DNA | 5 |
| Pteridaceae | *Adiantum* (Unidentified) |  | DNA | 5 |
| Dryopteridaceae | *Pleocnemia* (Unidentified) |  | DNA | 5 |
| Gleicheniaceae | *Dicranopteris* (Unidentified) |  | DNA | 5 |
| Thelypteridaceae | (Unidentified) |  | DNA | 5 |
| Cyatheaceae | (Unidentified) |  | DNA | 5 |
| Lejeuneaceae | (Unidentified) |  | DNA | 5 |

a = Type of detection (Fl = sighted on and/or caught near flowers, P = pollen found in faeces and/or on body, Fr = caught near fruiting trees, DNA=DNA metabarcoding)

**References**

Bumrungsri, S., Lang, D., Harrower, C., Sripaoraya, E., Kitpipit, K., & Racey, P. A. (2013). The dawn bat, *Eonycteris spelaea* Dobson (Chiroptera: Pteropodidae) feeds mainly on pollen of economically important food plants in Thailand. *Acta Chiropterologica*, 15(1), 95-104. doi:10.3161/150811013X667894

Hodgkison, R., Balding, S. T., Zubaid, A., & Kunz, T. H. (2004). Temporal variation in the relative abundance of fruit bats (Megachiroptera: Pteropodidae) in relation to the availability of food in a lowland Malaysian rain forest. *Biotropica*, 36(4), 522-533.

Start, A. N., & Marshall, A. G. (1976). Nectarivorous bats as pollinators of trees in West Malaysia. In *Tropical trees: variation, breeding and conservation* (pp. 141–149). London: Academic Press.

Thavry, H., Cappelle, J., Bumrungsri, S., Thona, L., & Furey, N. M. (2017). the diet of the cave nectar bat (*Eonycteris spelaea* dobson) suggests it pollinates economically and ecologically significant plants in Southern Cambodia. *Zoological Studies, 56*(17).
